# The effects of fitness self-testing with instant feedback on changes in health-related fitness among Chinese male college students

**DOI:** 10.1371/journal.pone.0342089

**Published:** 2026-02-24

**Authors:** Yongshun Wang, Xiaofen D. Hamilton, Rulan Shangguan, Anlu Yang, Na Xiao, Chenhao Wu, Sizhe Liu, Ren Yang, Jiren Zhang, Mark F. Hamilton

**Affiliations:** 1 College of Physical Education, Huaqiao University, Xiamen, China; 2 Department of Curriculum and Instruction, The University of Texas at Austin, Austin, United States of America; 3 School of Physical Education, South China University of Technology, Guangzhou, China; 4 Department of Physical Education, China University of Mining and Technology-Beijing, Beijing, China; 5 Department of Mechanical Engineering, The University of Texas at Austin, Austin, United States of America; University of Technology Sydney, AUSTRALIA

## Abstract

**Background:**

The decline in health-related physical fitness resulting from physical inactivity remains a critical global public health concern. Technology-supported fitness self-testing has the potential not only to improve students’ testing experiences but also to enhance their health-related fitness. However, the effectiveness of such approaches has not yet been systematically examined, and their validity within university populations remains largely unestablished.

**Method:**

A quasi-experimental research design with a control group (*n* = 45) and an experimental group (*n* = 44), incorporating pre- and post-tests, was employed in this study. The experimental group completed monthly self-testing sessions accompanied by GAI-generated instant feedback over a 16-week period, whereas the control group participated in general physical education classes that included multiple physical activities. Health-related fitness (HRF) was assessed using BMI, the one-mile run, pull-ups, and sit and reach tests. VO₂max was included as a covariate to control for baseline differences in HRF between the two groups. Repeated-measures multivariate analysis of covariance (RM-MANCOVA) was conducted to examine the effects of the intervention on HRF outcomes.

**Results:**

After controlling for baseline VO_2_max, RM-MANCOVA indicated significant time × group interaction for sit and reach (*p* < 0.001) and one-mile run (*p <* 0.05), with the intervention group demonstrating significant improvement in both tests. However, no significant differences were observed between groups for body mass index (BMI) and the pull-ups test.

**Conclusions:**

These findings suggested that HRF self-testing with instant GAI feedback was an effective intervention for improving certain HRF components, particularly flexibility and aerobic fitness. Further research is necessary to explore the long-term effects of self-testing and its application across diverse populations.

## Introduction

Health-related fitness (HRF) is a multidimensional construct encompassing essential components of physical fitness closely associated with health outcomes, including body composition, cardiorespiratory endurance, muscular strength and endurance, and flexibility [1–3]. Collectively, these components represent an individual’s ability to engage in physical activity (PA), which is crucial for mental health and overall well-being [4–6]. However, physical inactivity has been identified as a significant global health crisis [7]. A comprehensive analysis in *The Lancet Global Health* covering data from countries representing 96% of the world’s population reported that 27.5% of adults are insufficiently active [8], while the majority of adolescents do not meet the globally recommended PA guidelines [9], thereby jeopardizing their immediate and long-term health [10]. College students are in a critical transition period toward adulthood, a stage where establishing regular PA habits is essential for their long-term overall health [11]. A study indicated that, when controlling for BMI, Chinese college freshmen exhibited the poorest physical fitness index results, suggesting that this group has not yet established regular exercise behaviors [12]. Furthermore, research has revealed a higher prevalence of obesity among Chinese male college students, which may adversely affect their physical fitness performance. This gender disparity may be attributed to lower participation rates in structured PA, higher prevalence of sedentary leisure activities, and greater susceptibility to unhealthy weight gain among male college students compared with their female counterparts [8,13]. Targeting this population is therefore crucial for early intervention, as improving HRF during the university years can help mitigate long-term health risks [14]. Studies have shown that lower levels of muscular fitness, cardiorespiratory fitness (CRF), and flexibility contribute to a higher risk of chronic diseases [15–18], including cardiovascular disease, high blood pressure, and insulin resistance [19–22]. In particular, low CRF has been found to adversely affect health-related quality of life [19,23,24], putting them at a higher risk level for poor health in the future. Thus, for Chinese college students, the necessity of maintaining adequate HRF cannot be overstated.

Regular HRF testing is shown to play a pivotal role in fostering lifelong engagement in PA, which is essential for sustained health benefits [4,25]. HRF testing has the potential to motivate students to participate in more HRF activities if it is done appropriately [26–29]. However, traditional HRF testing, typically conducted by teachers every year, is often time-consuming and can cause student discomfort [30–32]. The public nature of traditional HRF assessments [33], combined with a lack of individualized evaluation [34] and immediate feedback [30,35], often contributes to lower engagement levels among students [36]. Therefore, HRF testing in PE programs should prioritize health monitoring, align with educational goals, and encourage self-determined motivation to support students’ health behaviors effectively [37].

It is important to point out that student-centered fitness self-testing presents a promising alternative to conventional HRF assessment methods [36,38]. It enables individuals to independently assess their HRF at any time and place, typically with the aid of technology [36,39]. The integration of technological devices such as fitness apps [40,41] and electronic testers enhances the accuracy and immediacy of feedback [38,42], allowing students to receive real-time updates on their fitness levels [36,39,43]. This immediate, personalized feedback helps students better understand their HRF, set specific fitness goals, and monitor their progress [19,36,44].

Examining HRF among Chinese college students is especially relevant in light of the national PE curriculum, which extends from primary education through university [3,45]. University-level PE represents the final stage where interventions can effectively influence the HRF behaviors and attitudes of a large segment of young adults [46]. Previous studies, primarily grounded in Western contexts, suggest that student-centered fitness self-testing incentivizes students and cultivates positive attitudes [36]. However, the validity of this approach within higher education environments remains underexplored. Specifically, the paucity of data regarding Chinese college students limits the understanding of this method’s cross-cultural applicability. While prior research has shown the positive effects of fitness self-testing on HRF in various contexts, studies specifically targeting college students in China remain limited. In addition, most existing studies have not employed quasi-experimental designs, which are typically used to evaluate the effectiveness of interventions in educational settings where students were enrolled in intact classes and random assignment to experimental and control groups was not feasible [27,36,47,48]. Therefore, this study aims to fill this gap in the literature by examining the effects of fitness self-testing on HRF among Chinese male college students, providing valuable insights into how this intervention could influence their overall health outcomes.

Specifically, the purpose of the current study was to examine whether the autonomy, personalized, and instant feedback provided by fitness self-testing could lead to sustained improvements in HRF among college male students. It is hypothesized that HRF self-testing with instant feedback is expected to significantly improve HRF in male college students, as assessed by key indicators such as BMI, cardiovascular endurance, muscular strength and endurance, and flexibility [21,49].

## Materials and methods

The study was approved by the Institutional Review Board of the senior author’s university (Approval No. STUDY00004614). All participants were provided with written informed consent prior to participation. No personal identifiers were collected at any stage of the study. Additional information regarding the ethical, cultural, and scientific considerations specific to inclusivity in global research is included in the Supporting Information (S1 Checklist).

### Research design

**Fig 1:**
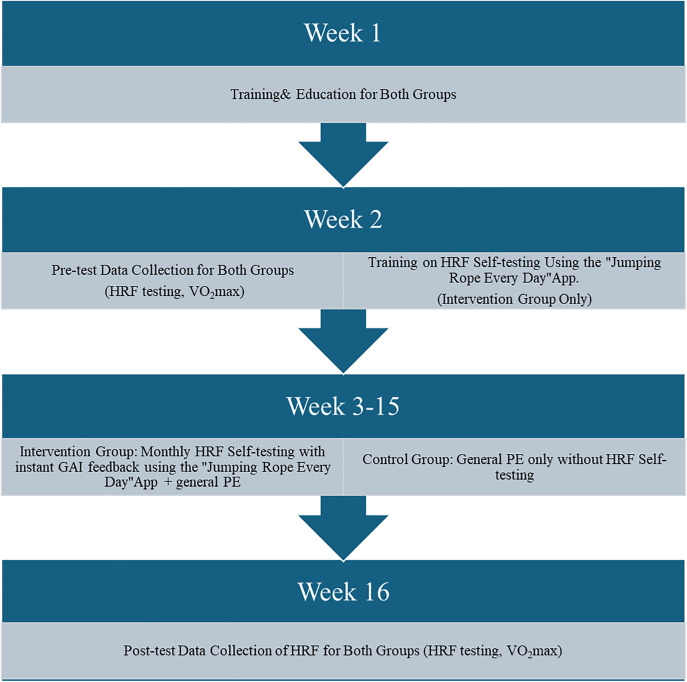
Experimental intervention process for fitness self-testing between the intervention group and the control group.

A quasi-experimental design incorporating pre- and post-tests with both control and intervention groups was utilized to assess the effects of fitness self-testing with instant generative artificial intelligence (GAI) feedback on HRF in Chinese male college students. In this study, the GAI component embedded in the mobile application was used to support movement recognition, automated scoring, and the provision of immediate, individualized feedback during selected HRF self-testing tasks. Participants in two general PE classes were selected, and the two classes were randomly assigned to the control group and the intervention group, respectively (see Fig. 1), as randomization at the individual level was not feasible due to administrative and instructional constraints within the university PE system; therefore, intact classes were used as the unit of assignment. Both groups had pre-and post-tests of their HRF conducted by the research team, while only the intervention group had the monthly fitness self-testing with instant GAI for their fitness exercise. The recruitment of participants occurred at the beginning of the semester and the intervention lasted for 16 weeks. The intervention consisted of monthly fitness self-testing with instant GAI feedback based on the testing results. VO₂max at the pre-test served as a covariate to account for initial differences between groups.

### Participants

The students enrolled in two general PE classes taught by the senior researcher took part in the study. Students in the two classes were informed about the study and invited to voluntarily participate. After one of the two classes was randomly assigned as the control group, the other class served as the experimental group with 47 individuals in each class initially. A Physical Activity Readiness Questionnaire (PAR-Q) [50] was also administered to screen for pre-existing injurie and only those with acceptable health were included at the beginning of the study. Participants were operationally defined as “healthy” if they reported no contraindications on the PAR‑Q and no self‑reported cardiovascular, respiratory, or musculoskeletal conditions that would limit moderate‑to‑vigorous physical activity. Throughout the study, three participants in the intervention group withdrew due to COVID-19, and two participants in the control group failed to complete the post-intervention assessment. Consequently, the final sample comprised 44 participants in the intervention group and 45 in the control group. All participants were residents on campus without a part-time job. The participants’ ages ranged from 18 to 19 years, and all were regular students, not PE students or student athletes.

### Materials

The “Jumping Rope Every Day” APP (Version 3.2.1), which is widely used in Chinese school PE, served as the core tool for the intervention of fitness self-testing with instant GAI feedback. Specifically, the APP allows participants to input their height and weight for automatic BMI calculation and utilizes advanced AI technology for precise movement data capture. It provides video-guided self-testing modules for four key HRF indicators: sit-and-reach (flexibility), one-mile run (aerobic fitness), pull-ups (upper body strength), and BMI (body composition). After each test, the APP generates personalized feedback, including test results, targeted exercise guidance, and a customized fitness prescription (e.g., addressing strengths/weaknesses in fitness components), creating a “Test-Learn-Practice-Evaluate” feedback loop [51]. This APP aligns with the data-driven trends in PE [52] and supports the research’s goal of enhancing HRF through personalized and accessible self-testing [37]. The APP supported both indoor and outdoor fitness practice and utilized GAI to evaluate exercises such as sit-and-reach. Based on the testing results, it provided instant feedback and automatically generated individualized exercise prescriptions to guide participants in adjusting their PA plans [51]. In the current study, participants in the experimental group were required to conduct their own fitness tests, evaluate their results, create personalized PA plans based on their testing outcomes, implement these plans, and continuously adjust their PA levels based on monthly self-testing results.

#### Reliability and validity of the app.

Prior to the intervention, the research team conducted a small-scale reliability check of the app because published evidence on its measurement properties was not available. Ten randomly selected participants were assessed simultaneously by the app and by experienced PE instructors using manual methods. To examine consistency between the two approaches, a two-way mixed-effects intraclass correlation coefficient (ICC) was calculated, yielding a value of 0.92. This level of agreement is generally interpreted as excellent [53]. Across all HRF indicators, the average discrepancy between app and instructor-derived scores was within 3%, suggesting that the app provides dependable measurements. On this basis, it was judged to be an appropriate instrument for use in this study.

#### Interventions.

The intervention consisted of monthly HRF self-testing, with participants scheduling sessions at their convenience each month, utilizing the “Jumping Rope Every Day” app for HRF testing and instant GAI feedback. Each participant in the intervention group was required to schedule and complete their self-testing sessions independently, inputting their height and weight data, and performing the four HRF tests (i.e., sit-and-reach, one-mile run, pull-ups, and BMI) at their convenience. Specifically, for BMI, weight (kg) and height (cm) were measured using the school’s fixed stadiometer and weight scale, with participants standing barefoot on the device. Intervention group participants manually entered these values into the “Jumping Rope Every Day” App, which automatically calculated the BMI value. For flexibility, participants used the “Jumping Rope Every Day” App for the sit and reach (cm) AI test, which intelligently evaluated and scored the sit and reach scores. For the one-mile run (s), participants used the “Jumping Rope Every Day” App’s target run feature, input the target distance, and start running. The App automatically recorded the time used to run one mile. For upper body strength, participants used a high bar or horizontal bar specifically designed for the pull-ups test on campus, ensuring the bar’s thickness is suited for their hands. They recorded the number of pull-ups completed and were required to submit a video recording of their performance as verification. The results were then logged into an online collection form provided by the instructor and TAs. After completing each test, participants received instant GAI feedback from the App. This feedback included their performance metrics, standardized scores based on national fitness standards, and automated analysis of HR. Based on this information, the App provided targeted exercise suggestions tailored to specific HRF deficits (e.g., various flexibility exercises for those with low sit-and-reach scores). Participants were encouraged to adjust their PA plans using these suggestions. While they maintained autonomy in activity choice, the App offered structured guidance on training focus, intensity, duration, and frequency. It also generated personalized training sessions, typically comprising warm-up, targeted exercise modules, and cool-down components. The PE instructor and two teaching assistants (TAs) provided guidance and supervision throughout the intervention process. These self-test data were not included in the statistical analyses.

In contrast, the control group did not participate in fitness self-testing or receive instant GAI feedback through the App. They attended regular university PE classes as part of a general PE course. Because the two groups were drawn from different classes, control group participants were unaware of the experimental procedures and had no knowledge about the use of the App. Aside from the intervention, both groups received identical instruction in terms of content, class duration, instructional format, and instructor, with lessons focused on basic volleyball techniques, drills, and gameplay because the two classes were taught by the same instructor and TAs. No additional data were collected on the specific activities performed by the control group during the study period.

### Measures

#### HRF variables.

The HRF tests utilized in China comprised body mass index (BMI), sit-and-reach, one-mile run, and pull-ups for male students. Unlike the FitnessGram in the US and the ALPHA in the European Union, which are commonly implemented school-based fitness test batteries [54], sit-ups or curl-ups were not used for male HRF testing in China. Instead, pull-ups were used to test upper body muscular strength and endurance [3]. The same testing procedures were followed for the test items that were identical to those in FitnessGram or ALPHA.

These HRF variables refer to the standardized, instructor-administered pre- and post-tests conducted at fixed locations and scheduled times for both the intervention and control groups. These centrally organized assessments, supervised by PE instructors, served as the official outcome measures for statistical analyses in this study.

#### Covariate variables.

To account for potential baseline differences, VO₂ max was selected as a covariate in the analysis. VO₂ max, defined as the maximum rate of oxygen uptake during graded exercise, is recognized as the gold-standard measure of cardiorespiratory fitness VO₂max [55]. It integrates cardiovascular, respiratory, and muscular functions, providing a comprehensive index of an individual’s aerobic performance capacity. Higher VO₂max values indicate more efficient oxygen delivery and utilization, allowing for sustained exercise at greater intensities and more rapid post-exercise recovery [56]. Given its strong association with HRF and its sensitivity to both training adaptations and habitual activity levels, VO₂max was considered an essential control variable to minimize confounding influences when evaluating the effects of the intervention. No other covariates were included in the analyses.

### Procedure and intervention fidelity

As noted earlier, pre- and post-tests of HRF mandatorily used in China (i.e., BMI, one-mile run, pull-ups, and sit-and-reach) were administered at the beginning and end of the semester to collect data for the study (see Fig. 1). All assessments were conducted by a certified PE instructor and his TAs in accordance with the standardized HRF testing protocol mandated by China’s Department of Education.

#### Fidelity of interventions.

Intervention fidelity was maintained through multiple data sources. The “Jumping Rope Every Day” app supported the consistency of HRF testing by enabling instructors to store, track, and monitor all testing materials. All self-testing activities produced electronic records with timestamps, accessible to instructors, TAs, and participants. For example, for the sit-and-reach and pull-ups tests, the app’s GAI-based timing, counting, and video-recording functions were used throughout the testing process to verify accurate implementation. For the one-mile run, the app’s target run feature automatically calculated distance and time, with running time and trajectory data viewable only by instructors, TAs, and the participant.

### Data analyses

Descriptive statistics (means and standard deviations (*SD*)) were computed for all HRF variables, including BMI, sit-and-reach, one-mile run, and pull-ups, in both the pre-test and the post-test. Then, the assumptions for Repeated Measure Multivariate Analysis of Covariance (RM-MANCOVA) were performed. Preliminary analysis showed that scores of all testing items at pre-and post-tests were moderately correlated (Pearson’s *r* ranged between 0.34 and 0.69), substantiating the use of MANCOVA [47]. In addition, power analysis using G*Power suggested a total sample of 64 participants to achieve 0.80 power with a 0.05 alpha level and a medium effect size (*ES* = 0.25) for performing RM-MANCOVA with two measurements. The number of participants (*N* = 89) in this study met the recommended sample size. Therefore, RM-MANCOVA was performed using SPSS 26.0 to test the changes in overall HRF between the pre-and post-tests, after controlling for pre-test VO₂max. The four HRF variables served as within-subject dependent variables. Assumptions of linearity, multicollinearity, and normality were examined prior to the analysis. Box’s M was not significant [Box’s M = 50.09, *F*_(36, 25441)_ = 1.36, *p* < 0.001], indicating that the homogeneity of covariance matrices assumption was satisfied. Therefore, Wilks’ Lambda was used as the primary multivariate test statistic [57]. For significant RM-MANCOVA, post-hoc test univariate ANOVAs were conducted, and partial eta squared (η²) was calculated to assess the effect size (cut-off value of.001,.006 and.14 for small, medium, and large effect sizes, respectively) [47]. The Chi-square tests were performed to examine whether the distribution of BMI categories (i.e., underweight, normal weight, pre-obesity, and obesity) differed between the control and intervention groups at both pre-test and post-test stages. This analysis was conducted because mean BMI values in both groups were within the normal range at baseline, and categorical analysis allowed the detection of potential shifts in weight status that might not be reflected in mean values alone.

## Results

### BMI changes between pre- and post-test

The Chi-square test indicated no significant differences in BMI category distributions between the intervention and control groups at pre-test [*χ²*_(3,89)_ = 3.67, *p* = 0.30], indicating that the BMI of the groups was about the same before the intervention began. Similarly, the post-test analysis revealed no significant differences in BMI category distributions between the groups [*χ²*_(3,89)_ = 3.98, *p* = 0.26] (see Table 1).

**Table 1:** Percentage of BMI categories.

Classification	Pre-test		Post-test		*χ²*	*p*
	Control (%)	Intervention (%)	Control (%)	Intervention (%)		
Underweight (BMI < 18.5)	10 (11.2)	6 (6.7)	8 (9.0)	3 (3.4)	0.41	0.52
Normal (18.5 ≤ BMI < 24.9)	28 (31.5)	29 (32.6)	30 (33.7)	35 (39.3)	1.50	0.47
Overweight (25.0 ≤ BMI < 29.9)	5 (5.6)	10 (11.2)	5 (5.6)	7 (7.9)	0.07	0.79
Obesity (BMI ≥ 30.0)	1 (1.1)	0 (0)	1 (1.1)	0 (0)		
*χ²*	3.67		3.98			
*p*	0.30		0.26			

Note: Due to the small sample size for the obesity group, no Chi-square tests were performed.

### HRF changes between pre- and post-tests

A one-way RM-MANCOVA was performed to examine whether scores on HRF testing changed over time (pre vs. post) after controlling for their pre-test VO₂max. Four dependent variables were used: BMI, sit and reach, one-mile, and pull-ups. There was a significant main effect of time (pre- vs. post-) in the RM-MANCOVA with a large effect size [Wilks’ Λ = .81, *F*_(4, 83)_ = 4.96, *p* < 0.05, η^2^ = 0.21], indicating overall significant increases in HRF across the semester. The post-hoc tests indicated that there were significant increases in sit and reach and the effect size was large [*F*_(1,87)_ = 12.69, *p* < 0.001, η^2^ = .13] and one-mile run with a medium effect size [*F*_(1,87)_ = 8.36, *p* < 0.05, η^2^ = .03] (see Table 2).

**Table 2:** Difference between groups of male college students’ HRF.

Variable	Control (*N* = 44)		Intervention (*N* = 45)		Interaction effect (*F*)	
	Pre-test (*SD*)	Post-test (*SD*)	Pre-test (*SD*)	Post-test (*SD*)	Time	Time × group
**BMI**	21.06 (3.18)	21.64 (3.99)	22.38 (3.85)	23.04 (4.55)	0.14	0.01
**Sit and Reach**	11.16 (6.72)	16.09 (4.86)	7.25 (7.66)	18.40 (7.91)	12.69**	47.72**
**One-mile run**	10.42 (0.89)	9.30 (0.66)	10.35 (0.68)	8.77 (0.99)	8.36*	4.37*
**Pull-Ups**	4.48 (3.43)	5.86 (3.27)	5.78 (4.67)	8.51 (5.42)	0.26	3.96

Note:* indicated significant at *p* < 0.05, ** indicated significant at *p* < 0.01

### The difference between the Intervention and Control Groups in HRF changes

**Fig 2:**
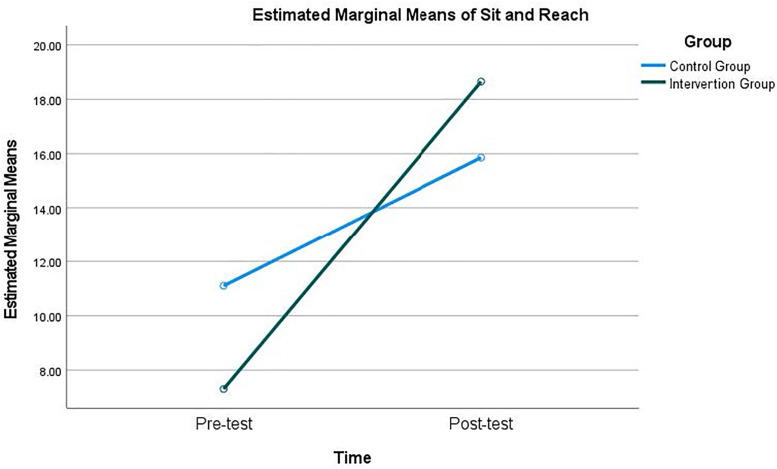
Pre- and post-test estimated sit and reach mean score of both intervention group and control group.

**Fig 3:**
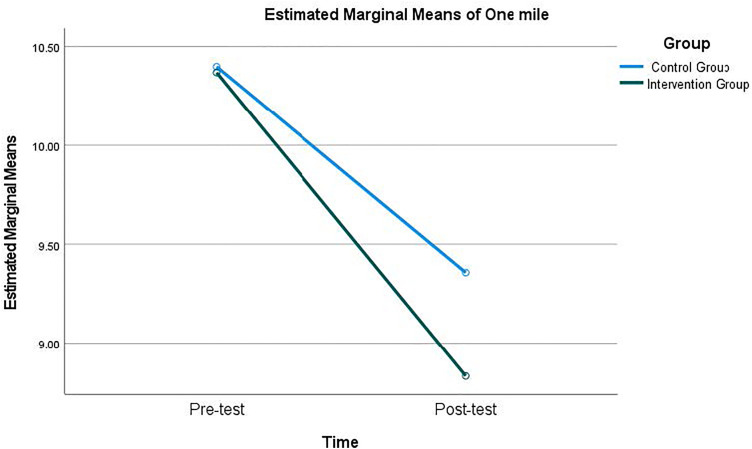
Pre- and post-test estimated one-mile run mean score of both intervention group and control group.

The RM-MANCOVA test revealed that there was a significant main effect of time × group interaction with a large effect size [Wilks’ Λ = .62, *F*_(4, 83)_ = 12.88, *p* < 0.001, η^2^ = 0.38], indicating that changes in HRF differed significantly between the intervention and control groups. Post-hoc univariate tests indicated a significant time × group interaction was found in sit and reach with a large effect size [*F*_(1,87)_ = 47.72, *p* < 0.001, η^2^ = .36], and one-mile run with a large effect size [*F*_(1,87)_ = 4.37, *p* < 0.05, η^2^ = .48], demonstrating that the intervention group improved more substantially than the control group. No significant interaction effects were found for BMI or pull-ups. Detailed values for each test are presented in Table 2 and visualized in Fig. 2 and 3.

## Discussion

This study set out to investigate the effects of fitness self-testing with instant feedback interventions on HRF among Chinese male college students. Although yearly required fitness testing has existed in PE programs for more than half a century [4,54,58], a significant increase in college students’ HRF remains to be seen [58–60]. This persistent educational and health-related issue has led to calls for enhanced HRF through school-based PE programs in China [58,61]. Moreover, it also raises questions about the necessity of regular fitness testing within these programs [27,62].

The findings from the current study demonstrated significant improvements in aerobic fitness and flexibility in the intervention group, contributing to the existing body of knowledge by highlighting the potential of self-testing as an effective means of enhancing fitness among college students [48]. As documented, college PE represents the last opportunity to improve the fitness levels of a substantial segment of emerging young adults. Therefore, further investigation into the relationship between college PE and student HRF is essential to promptly improve overall health [59]. This need is particularly pressing for Chinese students, who are required to undergo fitness tests annually throughout their four years of college [5,61]. Notably, no previous studies have explored the use of HRF self-testing among Chinese college students. Our study marks the first attempt to investigate an alternative approach to fitness testing through a student-centered lens, addressing some of the well-known flaws and barriers inherent in the traditionally teacher-centered methods.

Although self-testing is not a new concept in educational settings [63,64], it has not been widely and often used in school-based PE programs [36,39,48]. To date, to our knowledge, it is still unclear why HRF self-testing has not been popular among teachers in PE. The lack of research on the topic may be one of the reasons. As suggested by Keating and colleagues [36], with the assistance of new technologies for better reliability, validity, and accountability, HRF self-testing with instant GAI feedback may become more feasible in PE programs. The integration of technology via the “Jumping Rope Every Day” app may have catalyzed the success of HRF self-testing. This digital platform not only facilitated real-time feedback and progress tracking but also resonated with the technological proclivities of the student demographic. The app’s GAI-assisted features for exercises like sit-ups and sit and reach, along with its ability to record testing results, likely contributed to the heightened engagement and subsequent improvements in HRF [36,39]. Overall, using a quasi-experimental research design with standardized pre- and post-intervention HRF assessments administered by the research team, the present study identified significant improvements in flexibility and aerobic fitness among participants in the intervention group. It is important to note that the HRF self-testing activities were implemented as part of the intervention process rather than as outcome measures for statistical analysis. During the intervention, students in the experimental group obtained objective, instant feedback from AI-assisted self-testing, which enabled them to better understand their current fitness status, identify specific weaknesses, and adjust their PA plans accordingly. These iterative, self-regulated adjustments over the semester were subsequently reflected in the objectively measured HRF outcomes collected during the post-test by trained instructors. Taken together, the findings suggest that technology-assisted HRF self-testing may function as a behavioral catalyst, supporting students’ engagement in purposeful PA and being associated with measurable improvements in selected components of HRF.

It is important to acknowledge that both the intervention and control groups demonstrated improvements over time in selected HRF measures, as indicated by the significant main effect of time. This pattern suggests that regular participation in college PE classes and repeated exposure to standardized fitness testing across the semester may be associated with general performance gains, such as adaptation to routine PA, or heightened awareness of fitness expectations. However, the significant time × group interaction effects for sit and reach and the one-mile run, both with large effect sizes, indicate that improvements were significantly greater in the intervention group than in the control group. This pattern suggests that, beyond general time-related improvements, the self-testing with instant feedback intervention was associated with greater gains in these HRF components. These differential improvements are consistent with the tenets of self-determination theory, which posits that autonomy and personal investment are key drivers of behavioral change [47]. The intervention’s success in these areas suggests that the self-testing approach, by providing instant personalized feedback and fostering a sense of ownership over one’s fitness journey, can effectively engage students and motivate them to improve their performance [36,37]. The significant results may be explained, in part, by the frequency of fitness testing implemented in the PE program in which students were required to self-test their HRF each month with multiple trials if they were not satisfied with their testing results. This repeated, self-paced testing process may have been associated with lower perceived anxiety and stress. Because it was self-testing, the results of the testing were kept private, causing no public embarrassment even if students did not perform well. Another reason may be related to the connection between the testing results and behavioral changes. As noted in the literature, it was found that fitness testing results often were not used to educate students to be physically active and students did not understand why they needed to take the tests [34,65]. The requirement to make changes in PA behaviors based on their testing results in the intervention group each month may have helped students understand the usefulness of the monthly testing.

However, the lack of significant changes in BMI and pull-ups scores warrants further exploration. Existing literature suggests that the absence of significant changes in BMI and is likely multifactorial. First, the 16-week duration of this study, while effective for improving flexibility and aerobic capacity, may have been insufficient to elicit measurable changes in body composition and upper-body muscular strength, which often respond more slowly to general PA interventions. More critically, the intervention focused on self-testing and general activity promotion without incorporating structured resistance training or specific nutritional guidance, which are two critical factors to changes in BMI and pull-ups [16,22]. It is necessary to note that the average BMI values for both groups remained within the acceptable range from pre-test to post-test, indicating that no efforts are needed to reduce students’ BMI. Conversely, the disappointing performance in pull-ups, which showed no significant change despite a positive trend in the intervention group, raises concerns. Improvements in pull-ups performance typically require sustained, movement-specific practice and progressive overload, which are difficult to achieve through short-term, self-directed activity alone [66,67]. Behavioral and cognitive factors, such as low self-efficacy and the perceived difficulty of the movement [68], may have further limited adherence to strength-training recommendations. Furthermore, nutrition plays a critical role in strength development; unfortunately, no nutritional intervention was incorporated in this study, leaving the reasons for the lack of change in upper body muscular strength unclear [69].

There are limitations that are worth noting. First, the sample was homogeneous, consisting solely of male students enrolled in college PE classes, which are mandatory during the first two years of study in China. These participants may have had a higher baseline interest in fitness and may have been more motivated to use the app compared with typical male juniors and seniors, who are no longer required to take PE classes. No gender differences were explored, as the study was intentionally designed to examine effects in male students. Thus, it remains uncertain whether similar effects would be observed among female college students or non-PE majors. Third, the intervention was conducted over only one semester, which may have contributed to the insignificant changes in BMI and pull-ups. Fourth, the study was conducted at a southern university in China setting with a mild climate, which may have facilitated participants’ overall engagement in physical activity outside structured sessions (e.g., running or active commuting), as weather conditions can influence habitual activity levels beyond formal indoor exercises. Finally, no data were collected regarding the control group’s PA volume or their use of other exercise apps. As a result, outside variables might exist. Future research should aim to diversify the participant pool and explore the long-term effects of self-testing interventions in varied environments. Caution should be exercised when generalizing the results of this study to the broader population.

## Conclusion

HRF self-testing, supported by new technologies, has shown promising results among male college students in China, despite not all fitness components demonstrating improvement. This highlights the clear need for further research to explore the long-term impacts of self-testing interventions and to identify complementary strategies that can effectively address all aspects of HRF. Future studies should examine the influence of self-testing on a broader range of fitness components, particularly those that did not show significant changes in this study. Additionally, research investigating the role of self-testing across diverse cultural and educational contexts will be instrumental in refining and validating the effectiveness of this intervention approach on a global scale. Moreover, it is essential to explore potential gender differences in the effects of HRF self-testing, given that female students were not included in this study.

## Supporting information

S1 Checklist Inclusivity in global research questionnaire. (DOCX)

S1 File Minimal data set. (XLSX)
